# Expression of Concern: Mesenteric Panniculitis and COVID-19: A Rare Association

**DOI:** 10.7759/cureus.x50

**Published:** 2022-04-07

**Authors:** Israa A Alyousef, Zainab A Alsaileek, Manar A Alabdulsalam, Muhannad A Almohanna, Naif A Alshaqhaa, Mohammed M Alqahtani, Anas A Al Alyany, Muhannad A Alzahrani, Hawazin A Fallatah, Jalal I Alqadhib, Abdulaziz M Alhawsawi, Abdulelah D Alsuhaymi, Ali M Alasmari, Abdullah J Alshareef, Faisal Al-Hawaj

**Affiliations:** 1 College of Medicine, Dar Al Uloom University, Riyadh, SAU; 2 College of Medicine, Qassim University, Buraydah, SAU; 3 College of Medicine, Almaarefa University, Riyadh, SAU; 4 College of Medicine, Jordan University of Science and Technology, Irbid, JOR; 5 College of Medicine, Al-Baha University, Al-Baha, SAU; 6 College of Medicine, King Abdulaziz University, Jeddah, SAU; 7 College of Dentistry, Imam Abdulrahman Bin Faisal University, Dammam, SAU; 8 General Practice, Madinah General Hospital, Medina, SAU; 9 College of Medicine, Hunan Normal University, Changsha, CHN; 10 College of Medicine, Umm Al-Qura University, Mecca, SAU; 11 College of Medicine, Taif University, Taif, SAU; 12 College of Medicine, Imam Abdulrahman Bin Faisal University, Dammam, SAU

The concern relates to the provenance of this article as brought to our attention by Faisal Alhawaj, who denies authorship of this article and others published in Cureus. These articles were submitted and subsequently published purportedly as an effort coordinated by Imam Abdulrahman Bin Faisal University to ensure all medical interns publish at least one peer-reviewed article in order to qualify for enrollment in a postgraduate residency program as stipulated by The Saudi Commission for Health Specialties (SCFHS).

The journal has not been presented with enough evidence to warrant the formal retraction of these articles as both Imam Abdulrahman Bin Faisal University and The Saudi Commission for Health Specialties have failed to respond to numerous communications requesting additional information regarding these allegations. While we acknowledge that the provenance of these articles is very much in question, we cannot act until these claims have been investigated by the appropriate institutions with the results of said investigation communicated to Cureus.

The concern and this note will remain appended to the above-mentioned article until Cureus is provided with official confirmation from Imam Abdulrahman Bin Faisal University or The Saudi Commission for Health Specialties.

